# Elevated expression of lung development-related protein HSP90β indicates poor prognosis in non-small cell lung cancer through affecting the cell cycle and apoptosis

**DOI:** 10.1038/s41392-021-00465-y

**Published:** 2021-02-26

**Authors:** Xiang Wang, Yaru Wang, Lin Feng, Minghui Wang, Kaitai Zhang, Yousheng Mao, Ting Xiao, Shujun Cheng

**Affiliations:** 1 State Key Laboratory of Molecular Oncology, Department of Etiology and Carcinogenesis, National Cancer Center/National Clinical Research Center for Cancer/Cancer Hospital, Chinese Academy of Medical Sciences and Peking Union Medical College, Beijing, China; 2 Department of Thoracic Surgery, National Cancer Center/National Clinical Research Center for Cancer/Cancer Hospital, Chinese Academy of Medical Sciences and Peking Union Medical College, Beijing, China

**Keywords:** Lung cancer, Tumour biomarkers

**Dear Editor,**

Oncogenesis was considered to be similar to the early embryonic development process in a variety of ways.^1^ Using normal development samples as research models will help us understand tumors and identify potential biomarkers. Heat-shock protein 90 beta (HSP90β, *HSP90AB1*) is one of the major isoforms of HSP90 and is involved in embryonic development, signal transduction, and cellular adaptability. The aberrant expression of HSP90β was shown to be associated with lung cancer.^2^ Our previous work has identified that HSP90β could be used as a potential biomarker of lung adenocarcinoma (LUAD).^3^ Therefore, it is necessary to investigate the HSP90β in the occurrence and development of non-small cell lung cancer (NSCLC), which can provide new ideas for the diagnosis and treatment of NSCLC.

In our current study, we first carried out unsupervised hierarchical clustering analysis and principal component analysis to divide the nine time points of rhesus macaque lung development data^4^ into three phases with distinct features: Ph1, Ph2, and Ph3 (Fig. 1a and Supplementary Fig. S1a). To understand the biological significance in each phase, we performed gene coexpression analysis and GO (Gene Ontology) pathway analysis on 12,040 homologous genes. Gene coexpression analysis showed that different genes were highly expressed in the three different phases of lung development, named as cluster1 (5706 genes), cluster2 (4122 genes), cluster3 (2212 genes) (Supplementary Fig. S1b and Supplementary Table S1). The GO analysis suggested that Ph1 is associated with DNA replication, RNA splicing, and cell cycle, while Ph2 is mainly involved in focal adhesion, cell–substrate adhesion, and Ras protein signal transduction. Ph3 was the mature phase of the lung and genes were enriched in immune regulation pathways (Supplementary Fig. S1c). We then constructed a protein–protein interaction network for the genes involved in the cell cycle signaling pathway in Ph1 and found that HSP90β mediated the crosstalk between *AKT1* and *TP53* (Fig. 1b). The HSP90β was found specifically expressed at Ph1 and decreased at Ph2 and Ph3 (Supplementary Fig. S1d). This indicated that HSP90β is crucial at the early stage of lung development, suggesting that the eccentric expression of HSP90β may initiate and facilitate the growth of tumor. Besides, from our previously reported human lung microarray data,^5^ we found that throughout the sequential developmental of the lung to the cancer tissues, HSP90β expression first decreased and then increased, while the adult lung tissues and adjunct normal lung tissues exhibited the lowest expression. HSP90β was significantly higher (*P* < 0.001) in NSCLC tissues than that in adjacent normal lung tissues (Fig. 1c). We then examined the messenger RNA expression of HSP90β in 64 lung cancer tumor tissues and paired normal lung tissues and confirmed the higher expression of HSP90β in tumor tissues (Supplementary Fig. S1e).

**Fig. 1:**
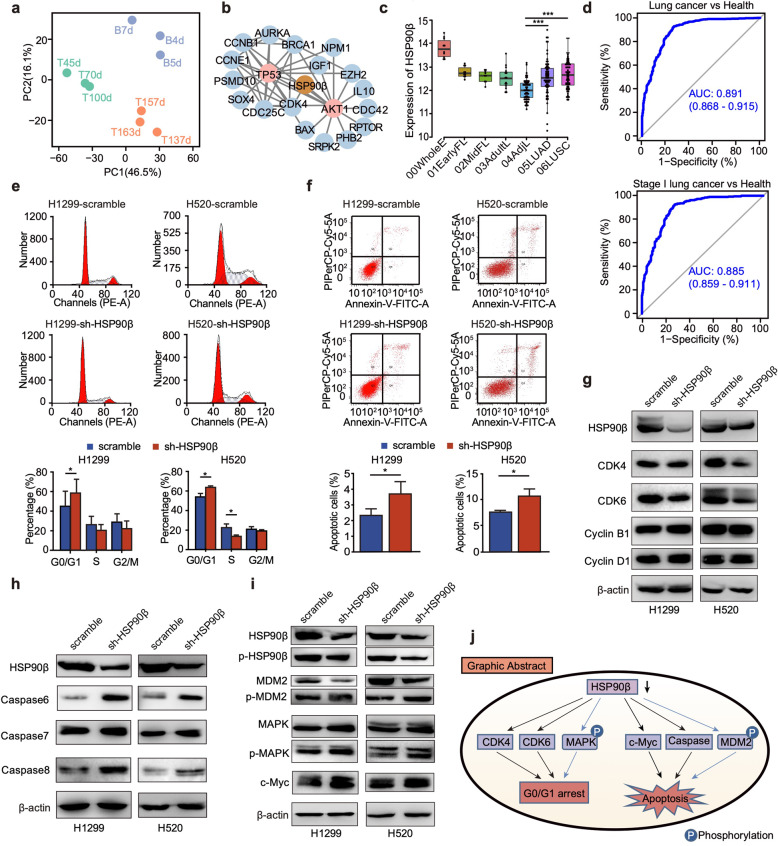
**a** PCA (principal component analysis) of temporal RNA-seq data to separate the whole rhesus macaque lung developmental process into three phases (Ph1: early stage, including T45d, T70d, T100d; Ph2: middle stage, including T137d, T157d, T163d; Ph3: late stage, including B4d, B5d, B7d). **b** Protein–protein interaction (PPI) network involved in cell cycle regulation identified at the Ph1. **c** The gene expression of HSP90β in four developmental stages of human lung tissues and lung cancer tissues (“00WholeE”: whole embryos at PWs 3–5, *n* = 10; “01EarlyFL”: lungs at 6–8 PWs, *n* = 10; “02MidFL”: lungs at 16–24 PWs, *n* = 9; “03AdultL”: adult lung tissues, *n* = 15; “04AdjL”: adjacent normal lung tissues, *n* = 60; “05LUAD”: LUAD tissues, *n* = 69; “06LUSC”: LUSC (lung squamous cell carcinoma) tissues, *n* = 69). Mann–Whitney *U* test was used to verify the significance between “04AdjL” and “05LUAD”, “04AdjL,” and “06LUSC.” **d** ROC (receiver-operating characteristic) curve of HSP90β in distinguishing NSCLC patients from healthy controls (upper panel) and ROC curve of HSP90β in distinguishing stage I NSCLC patients from healthy controls (lower panel, AUC: Area under the ROC curve. The 95% CI is indicated in parentheses). **e** Cell cycle of H1299 and H520 cells (top panel shows images of the cell cycle in the scramble and HSP90β knockdown cells, bottom panel shows the statistical column diagram (Student’s *t* test). **f** Cell apoptosis of H1299 and H520 cells (top panel shows images of the cell apoptosis, the bottom panel shows the statistical column diagram) (Student’s *t* test). **g** Western blot of cell cycle-related proteins in H1299 and H520 cells. **h** Western blot of cell apoptosis-related proteins in H1299 and H520 cells. **i** Western blot of several phosphorylated proteins involved in the cell cycle and apoptosis. **j** Graphical abstract of the effects of HSP90β on the cell cycle and apoptosis. ***P < 0.001; *P < 0.05

We further examined the plasma levels of HSP90β protein in 870 NSCLC patients (Supplementary Tables S2, S3, and S4). The correlation between HSP90β and clinical information of NSCLC patients is shown in Supplementary Tables S5 and S6. The concentration of HSP90β in the plasma of NSCLC patients was significantly higher (*P* < 0.001) than that in healthy persons (Supplementary Fig. S2a), and lung squamous cell carcinoma patients was significantly higher (*P* < 0.001) than that in LUAD patients (Supplementary Fig. S2b). We then assessed the diagnostic impact of HSP90β on NSCLC. When distinguishing NSCLC patients from healthy controls, the cutoff value of the HSP90β protein in plasma was 54.22 ng/ml, the sensitivity of the diagnosis of NSCLC was 92.9%, the specificity was 73.1%, and the area under the receiver-operating characteristic curve was 0.891(95% confidence interval = 0.868–0.915; Fig. 1d). While distinguishing early NSCLC patients (stage I) from healthy controls, the area under the receiver-operating characteristic curve was 0.885 (95% confidence interval = 0.859–0.911; Fig. 1d). The relationship between HSP90β and the prognosis of 733 NSCLC patients was also analyzed. HSP90β were significantly associated with disease-free survival (Supplementary Fig. S2c) and overall survival (Supplementary Fig. S2d) in NSCLC patients. Higher HSP90β levels in patients indicated a poorer prognosis.

Since HSP90β highly expressed in lung cancer, to further verify its role in the cancer cell, we knocked down HSP90β in two NSCLC cell lines, H1299 and H520. The protein and messenger RNA levels showed that silencing HSP90β was significant (*P* < 0.05) in the cell lines (Supplementary Fig. S3a, b). Both CCK8 and colony formation assays showed that the growth rate of the lung cancer cells was significantly decreased (*P* < 0.05) after HSP90β was knocked down (Supplementary Fig. S3c, d). This was further confirmed in vivo through nude mice model (Supplementary Fig. S3e). To explore the mechanism of tumor growth inhibition, we next evaluated the effect of HSP90β on the cell cycle and apoptosis in the cell lines described above. Silencing HSP90β expression significantly increased (*P* < 0.05) the number of H1299 and H520 cells in the G0/G1 phase and decreased in the S and G2/M phases (Fig. 1e). Cell apoptosis was assessed and revealed an increase in apoptotic cells in both cell lines after HSP90β silencing (Fig. 1f). We further evaluated the expression of proteins related to the cell cycle and apoptosis. Regarding those involved in the cell cycle, we found that CDK4 and CDK6 reduced, while cyclin D1 and cyclin B1 moderately upregulated in HSP90β-knockdown cells (Fig. 1g). Silencing HSP90β also increased the expression of cell apoptosis-related proteins c-Myc, caspase-6, caspase-7, and caspase-8 (Fig. 1h, i).

To further investigate the molecular mechanism by which HSP90β affects the cell cycle and apoptosis, a phospho-specific antibody microarray was used to examine differences between H1299 control and HSP90β-knockdown cells. By using a cutoff ratio of 1.12, we identified 53 differentially phosphorylated sites in multiple proteins (Supplementary Table S7). Among them, 17 phosphorylated proteins participate in the cell cycle and apoptosis (Supplementary Fig. S4a). We then determined the key branch signaling pathway of these phosphorylated proteins to clarify their relationships and regulations (Supplementary Fig. S4b). By using Western blotting, we confirmed the phosphorylation status of HSP90β (Ser^255^), mitogen-activated protein kinase (MAPK) (ERK1/2, Thr^202/204^), and MDM2 (Ser^166^). In the cell cycle pathway, the level of phosphorylated MAPK increased upon HSP90β knockdown coupled with the augment of MAPK protein (Fig. 1i). Higher expression of phosphorylated MDM2 was confirmed by Western blotting upon HSP90β knockdown (Fig. 1i), which is consistent with the phospho-specific antibody microarray. MDM2 is an oncogene and is essential for mediating apoptosis. Lower MDM2 expression was also observed after knockdown of HSP90β. Therefore, silencing HSP90β in lung cancer cells led to G0/G1 arrest through phosphorylated MAPK and resulted in the downregulation of CDK4 and CDK6, whereas the decreased HSP90β expression in lung cancer cells increased apoptosis through the upregulation of c-Myc, caspase-6, caspase-7, and caspase-8, as well as phosphorylated MDM2 (Fig. 1j).

In summary, we identified the vital role of HSP90β in NSCLC from transcriptome data of rhesus macaque lung tissues and human lung microarray data. We further determined the higher plasma level of HSP90β in NSCLC patients than that in healthy persons, providing the possibility of being used as a biomarker for screening and early detection as well as prognosis prediction of NSCLC patients. Our research also suggests that HSP90β affects apoptosis and the cell cycle in NSCLC cells by phosphorylating key proteins involved in multiple pathways, which would benefit the development of the targeted drug.

## Supplementary information

Table S5.Clinical information of LUSC and LUAD patients

Table S4.Healthy control HSP90β expression

Table S3.LUAD HSP90β expression

Table S2.LUSC HSP90β expression

Table S1.Genes enriched in three phases of rhesus macaque lung development

Supplementary materials

## References

[1] Ma Y (2010). The relationship between early embryo development and tumourigenesis. J. Cell Mol. Med..

[2] Biao R (2012). Upregulation of Hsp90-beta and annexin A1 correlates with poor survival and lymphatic metastasis in lung cancer patients. J. Exp. Clin. Cancer Res..

[3] Xu JY (2020). Integrative proteomic characterization of human lung adenocarcinoma. Cell.

[4] Yu X (2016). Crosstalk of dynamic functional modules in lung development of rhesus macaques. Mol. BioSyst..

[5] Feng L (2014). Gene expression profiling in human lung development: an abundant resource for lung adenocarcinoma prognosis. PLoS ONE.

