# Supplementary material for: Elevated expression of lung development-related protein HSP90β indicates poor prognosis in non-small cell lung cancer through affecting the cell cycle and apoptosis
Source: Signal Transduct Target Ther. 2021 Feb 26;6:82. doi: 10.1038/s41392-021-00465-y (PMC7907212; doi:10.1038/s41392-021-00465-y)
Supplement: Supplementary file 6 — Supplementary materials [file 41392_2021_465_MOESM6_ESM.docx]

Supplementary Materials for

**Elevated Expression of Lung Development-related Protein HSP90β Indicates Poor Prognosis in Non-Small-Cell Lung Cancer Through Affecting the Cell Cycle and Apoptosis**

Xiang Wang^1,#^, Yaru Wang^1,#^, Lin Feng^1^, Minghui Wang^1^, Kaitai Zhang^1^, Yousheng Mao^2, *^, Ting Xiao^1, *^, Shujun Cheng^1, *^

^1^State Key Laboratory of Molecular Oncology, Department of Etiology and Carcinogenesis, National Cancer Center/National Clinical Research Center for Cancer/Cancer Hospital, Chinese Academy of Medical Sciences and Peking Union Medical College, Beijing 100021, China

^2^Department of Thoracic Surgery, National Cancer Center/ National Clinical Research Center for Cancer/Cancer Hospital, Peking Union Medical College & Chinese Academy of Medical Sciences, Beijing 100021, China

#These authors contributed equally

* Correspondence e-mail: maoysherx@qq.com to Yousheng Mao

xiaot@cicams.ac.cn to Ting Xiao

chengshj@cicams.ac.cn to Shujun Cheng

**This PDF file includes:**

Materials and Methods

Supplementary Text

Figures. S1 to S4

Tables S6 to S7

Materials and Methods

**Transcriptome data of rhesus macaque lung tissues**

Transcriptome data of gene expression profiles for rhesus macaque lung tissues at different developmental phases were obtained from our published data with the raw sequencing data accession number SRP068754 in SRA (NCBI) data. Different developmental phases of lung tissues included Ph1 (45–100 gestational days, n=3, the sample in 100 days is the early sequencing data of our laboratory under the same conditions), Ph2 (137–163 gestational days, n=3), Ph3 (after birth at 4–7 days, n=3) were collected from rhesus macaques raised at the Institute of Laboratory Animal Science, Chinese Academy of Medical Sciences (CAMS) & Peking Union Medical College (PUMC). For the analysis of the transcriptome data, the expression level was normalized by per kilobase of exon region per million mappable (RPKM) reads, and only those genes expressed in more than three-quarters of samples were retained.

**Microarray data collection and processing**

Microarray data of gene expression profiles for human lung tissues at different developmental stages and for lung cancer tissues were obtained from our published data on the Gene Expression Omnibus (GEO) website under the accession numbers GSE43767, GSE40588 and GSE67061. Different developmental stages of lung tissues included whole embryos at postovulatory weeks (PWs) 3 to 5 (“00WholeE”; n = 10), lungs at 6 to 8 PWs (“01EarlyFL”; n = 10), lungs at 16 to 24 PWs (“02MidFL”; n = 9), and adult lung tissue samples (“03AdultL”; n = 15). Adjacent normal lung tissue samples (“04AdjL”; n = 60) were collected from primary lung cancer patients. LUAD tissue samples (“05LUAD”; n=69) and lung squamous cell carcinoma (LUSC) tissue samples (“06LUSC”; n=69) were collected from patients at the Cancer Hospital of the Chinese Academy of Medical Sciences & Peking Union Medical College. For data analysis, all the raw data were normalized by the median scale method using the R package “limma” (www.r-project.org). Screening probes represented the same gene, and only the probe showing the greatest mean intensity was retained.

**Clinical samples**

Peripheral blood samples were collected at the Cancer Hospital of the Chinese Academy of Medical Sciences & Peking Union Medical College between July 2007 and August 2013. Of the 870 non-small-cell lung cancer patients, 371 were LUSC patients and 499 were LUAD patients. For the LUSC cohort, 343 were males, 28 were females, and the mean age was 60 years. 234 patients in this cohort had the post-hospital follow-up visit (Supplementary Table 2). For the LUAD cohort, we use the published 499 patients data in our further analysis. The clinical information includes age, gender, tumor differentiation, TNM stage, lymph nodes metastasis (LNM), smoking status, family history of tumor and prognosis information which were listed in the Supplementary Table 3, 4 and 5. The median follow-up time for the patients was 48 months. Overall survival (OS) and disease-free survival (DFS) were given to evaluate the clinical outcomes. Exclusion criteria of the patients included the patients who had previous anti-cancer treatment with radiotherapy or chemotherapy or simultaneously suffering from other cancers. All patients signed a written informed consent before surgery, and the treatments were performed in accordance with present ethical principles. Peripheral blood samples were collected prior to surgery by venipuncture and preserved in EDTA-coated tubes. Samples were centrifuged at 4°C for 10 minutes at 1000 g to separate plasma from blood cells. Supernatants were collected, divided into aliquots, and stored at -80°C until use.

**Enzyme-linked immunosorbent assays (ELISAs)**

Plasma protein concentrations were assessed by ELISAs according to the manufacturer’s instructions. ELISA kits for HSP90β were purchased from USCN, China. In brief, 100 μl of diluted plasma was added to wells of an anti-HSP90β microplate and incubated at 37°C for 2 hours. Then, 100 μl of a prepared biotinylated HSP90β detector antibody was added to each well and incubated at 37°C for 1 hour. After 3 washes, 100 μl of the prepared conjugate was added to each well and incubated at 37°C for 1 hour. After 5 washes, the absorbance at 450 nm was measured immediately using a microplate reader (Bio-Rad Laboratory, Hercules, CA, USA).

**Cell culture and transfections**

Human NSCLC cell lines H1299 and H520 were purchased from the American Type Culture Collection (ATCC, Manassas, VA) and cultured in RPMI-1640 medium supplemented with 10% FBS (Gibco, Grand Island, NY, USA) at 37°C with 5% CO2. All cell lines were authenticated by short tandem repeat DNA proﬁling (Microread Gene Technology, Beijing, China). H1299 and H520 cancer cells were transfected with the *HSP90AB1* human shRNA plasmid (ORIGENE, Rockville, MD) using Lipofectamine 3000 according to the manufacturer's recommendations (Invitrogen, CA, USA). The OriGene pRS plasmid was used as retroviral shRNA vector. The OriGene pRS plasmid contains both 5’ and 3’ LTRs of Moloney murine leukemia virus (MMLV) that flank the puromycin marker and the U6-shRNA expression cassette. All inserts have the sequence structure: U6 promoter – GATCG -- 29 nt sense –TCAAGAG – 29 nt reverse complement --TTTTTT (termination) – GAAGCT. Two days after the transfection, the cells were trypsinized and seeded into culture medium supplemented with puromycin (Abcam, Cambridge, MA, USA, 2 µg/ml for H1299 cell lines or 0.3 µg/ml for H520 cell lines).

**RNA extraction and quantitative RT-PCR assays**

Total RNA was extracted from tissues or cultured cells using TRIzol reagent (Ambion, Life Technologies, USA) according to the manufacturer's instructions. RNA (1 µg) was reverse transcribed in a ﬁnal volume of 20 µl using random primers under standard conditions recommended by the PrimeScript RT Reagent Kit (Takara). Real-time PCR analyses were performed with SYBR Premix Ex Taq (Takara). The sense and antisense primers were synthesized as follows:

*HSP90AB1* forward 5’-AGAAATTGCCCAACTCATGTCC-3’,

*HSP90AB1* reverse 5’-ATCAACTCCCGAAGGAAAATCTC-3’,

18S forward 5’-TGCATGGCCGTTCTTAGTTG-3’,

18S reverse 5’-AGTTAGCATGCCAGAGTCTCGTT-3’.

Quantitative RT-PCR and data collection were conducted on an ABI 7500 real-time PCR system (Applied Biosystems). The 2 ^−ΔΔCt^ method was used to determine the relative quantitation of gene expression levels.

**Western blot assay and antibodies**

H1299 and H520 cancer cells were lysed on ice using RIPA lysis buffer (Applygen, China) supplemented with Halt™ Phosphatase Inhibitor Cocktail (Thermo Fisher Scientific, USA) and quantified using the Pierce BCA Protein Assay Kit (Thermo Fisher Scientific, USA). Equal amounts of the cell protein lysates were separated by 10% SDS-PAGE and transferred to a 0.25-μm polyvinylidene difluoride (PVDF) membrane (Millipore, Bedford, MA, USA). After being blocked with 10% nonfat dry milk in phosphate-buffered saline and 0.1% Tween 20 solution, membranes were incubated overnight at 4°C with the following primary antibodies: HSP90β (ABGENT, China, 1:1000), CDK4, CDK6 (Proteintech, USA, 1:1000), cyclin B1, cyclin D1, c-Myc (Abcam, USA, 1:1000), MAPK, caspase-6, caspase-7, caspase-8 (Cell Signaling Technology, USA, 1:1000), p-MDM2, p-MAPK (Cell Signaling Technology, USA, 1:500), MDM2, p-HSP90β (Abclonal, China, 1:500), and β-actin (Applygen, China, 1:5000). Then, the membranes were incubated with the appropriate secondary antibody for 1 hour at room temperature. The specific bands were detected using an ECL detection kit and an ImageQuant LAS 4000 mini system (GE Healthcare, NJ, USA).

**Cell proliferation assays**

A Cell Counting Kit-8 (CCK8, DOJINDO, Japan) assay was conducted according to the manufacturer's procedure. Briefly, the cultured cells were harvested, counted (2,000 cells/well) and cultured in 96-well plates at 37°C with 5% CO2. Four hours later, CCK8 solution was added to each well, and each well was measured spectrophotometrically at 450 nm after incubating for 2 hours. We consider this to be day “0” of the proliferation curve. Then, every 24 hours, cell growth was detected in the same manner. We detected cell proliferation for four days after day 0. The experiments were independently repeated three times.

**Colony formation assay**

Cells were harvested and counted (500 cells/well) in 60 mm cell culture dishes and cultured in 4 mL complete medium for 2 weeks. Then, the cells were ﬁxed with cold methanol for 30 minutes and stained with 0.1% crystal violet. The number of colonies was then counted.

**Flow cytometric analysis**

H1299 and H520 cancer cells were harvested 48 h after transfection. For cell cycle analysis, the cancer cells were ﬁxed with 75% ethanol overnight at 4°C. Then, the cells were washed three times with cold PBS and stained with 500 µl propidium iodide (PI, BD Pharmingen™, USA) in each tube at room temperature for 15 minutes before detection. Cell apoptosis was assessed according to the manufacturer's procedure detailed in the FITC Annexin V Apoptosis Detection Kit I (BD Pharmingen™, USA).

**Tumor xenograft model**

All the animal experiments were approved by the Institutional Animal Care and Use Committee at the Cancer Hospital of the Chinese Academy of Medical Science. Six-week-old female BALB/c nude mice were purchased from Beijing Vital River Company. For the experiment, 3×10^6^ lung cancer cells were injected into the right ﬂanks of nude mice in 100 µl sterile PBS to generate two groups: the scramble group (H1299 cells transfected with the scramble shRNA plasmid) and the knockdown group (H1299 cells transfected with the HSP90β shRNA plasmid). The mice were weighed every 3 days and were sacrificed about a month later, and the tumor tissues were obtained and measured.

**Phospho-protein antibody microarray profiling**

The Cancer Signaling Phospho Antibody Microarray CSP100 was used to further reveal the mechanisms by which HSP90β inﬂuences H1299 lung cancer cells. CSP100 contains 269 antibodies (Full Moon Biosystems, Sunnyvale, CA), and every antibody has six technical replicates. The phospho-protein antibody microarray experiment was conducted and analyzed by Wayen Biotechnology (Shanghai, China). The fluorescence intensity of each antibody was scanned with a GenePix 4000B (Axon Instruments, USA), and GenePix Pro 6.0 software was used to obtain the raw data. The phosphorylation ratio in one sample was calculated as follows: phospho ratio = phospho value/unphospho value; while in different samples, phospho ratio = phospho ratio_experiment_/phosphor ratio_control_.

**Statistical analysis**

Statistical analysis and data visualization were performed using the statistical software R version 3.6.0 packages “limma”, “ggplot2”, “survival”, “pROC” and “survminer”, “pheatmap”, “clusterProfiler”, “biomaRt”. GraphPad Prim 6.0 and Microsoft Office Excel 2016 were also used in the data analysis. BioMart was used to convert rhesus macaque genes into human homologous genes. Unsupervised hierarchical clustering (HC) analysis and Principal component analysis (PCA) were performed on 12,040 genes and grouped nine developmental time points into three phases. Gene co-expression analysis was conducted by Ward’s hierarchical clustering analysis. Gene Ontology (GO) term enrichment analysis was based on DAVID Bioinformatics Resources. STRING (<http://string-db.org>) and Cytoscape 3.7.1 were used to construct protein-protein interaction (PPI) network. The Kruskal-Wallis test was used to compare different groups of human lung developmental stages and different subtypes of lung cancer. The Kaplan-Meier estimation method was used for overall survival analysis, and a log-rank test was used to compare differences. The differences between lung cancer tissues and matched normal lung tissues were assessed through a two-tailed paired t-test. The differences between sh-HSP90β and scramble cells were assessed through a two-tailed paired t-test. *P* < 0.05 was considered statistically significant.


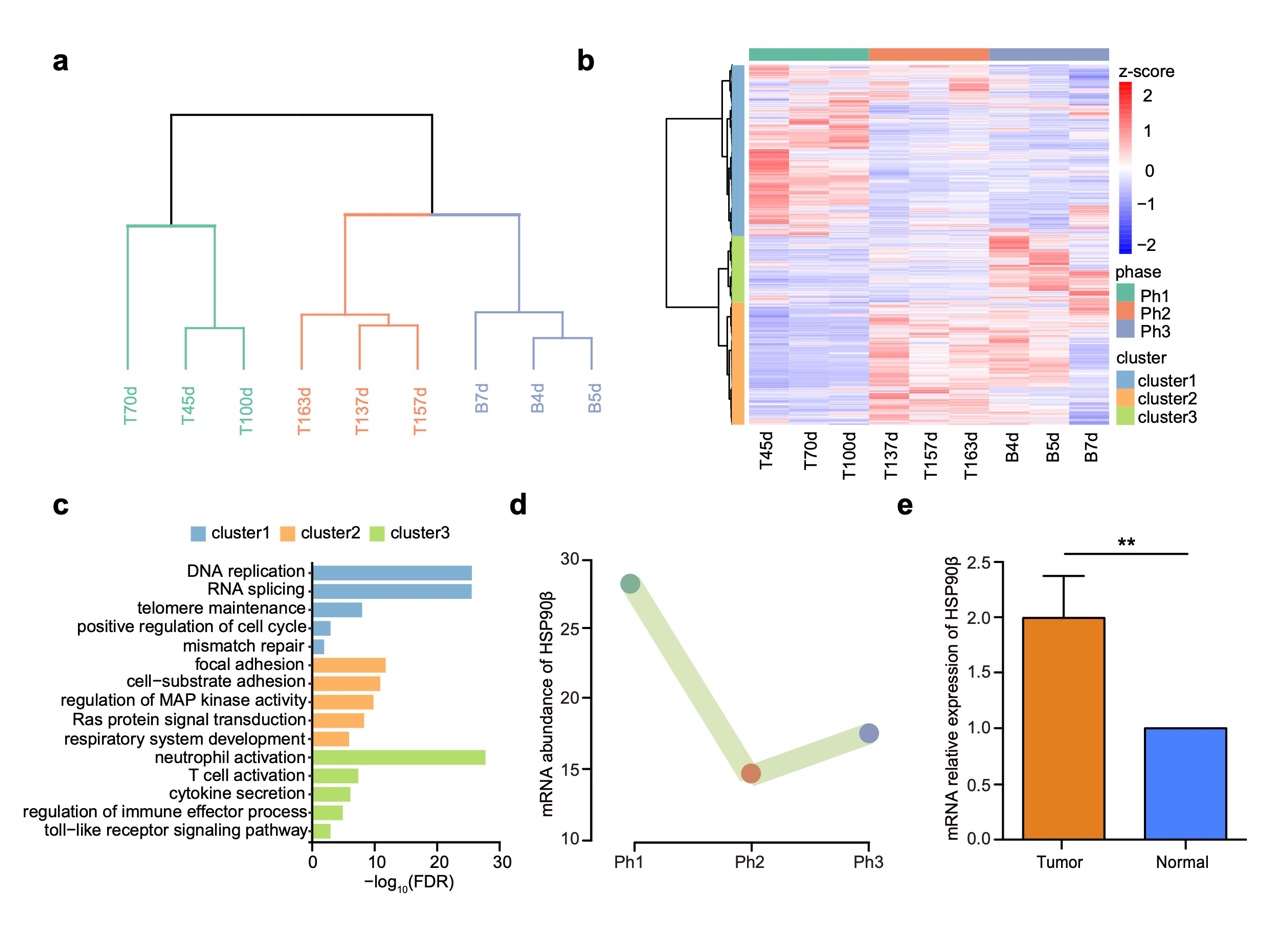


Figure. S1.

**Three developmental phases of rhesus macaque lung tissues and their biological features.**

**a** Hierarchical clustering of temporal RNA-seq data to separate the whole rhesus macaque lung developmental process into three phases. **b** Gene co-expression analysis of 12,040 homologous genes by Ward’s hierarchical clustering. **c** GO pathway analysis of three gene clusters. **d** Protein-protein interaction (PPI) network involved in cell cycle regulation identified at the Ph1. The median value of HSP90 expression in the three groups was used to represent the HSP90 expression level of the group. **e** The mRNA expression of HSP90β in lung cancer tissues and paired normal tissues (n = 64).Two-paired t-test, **, *P* < 0.01.


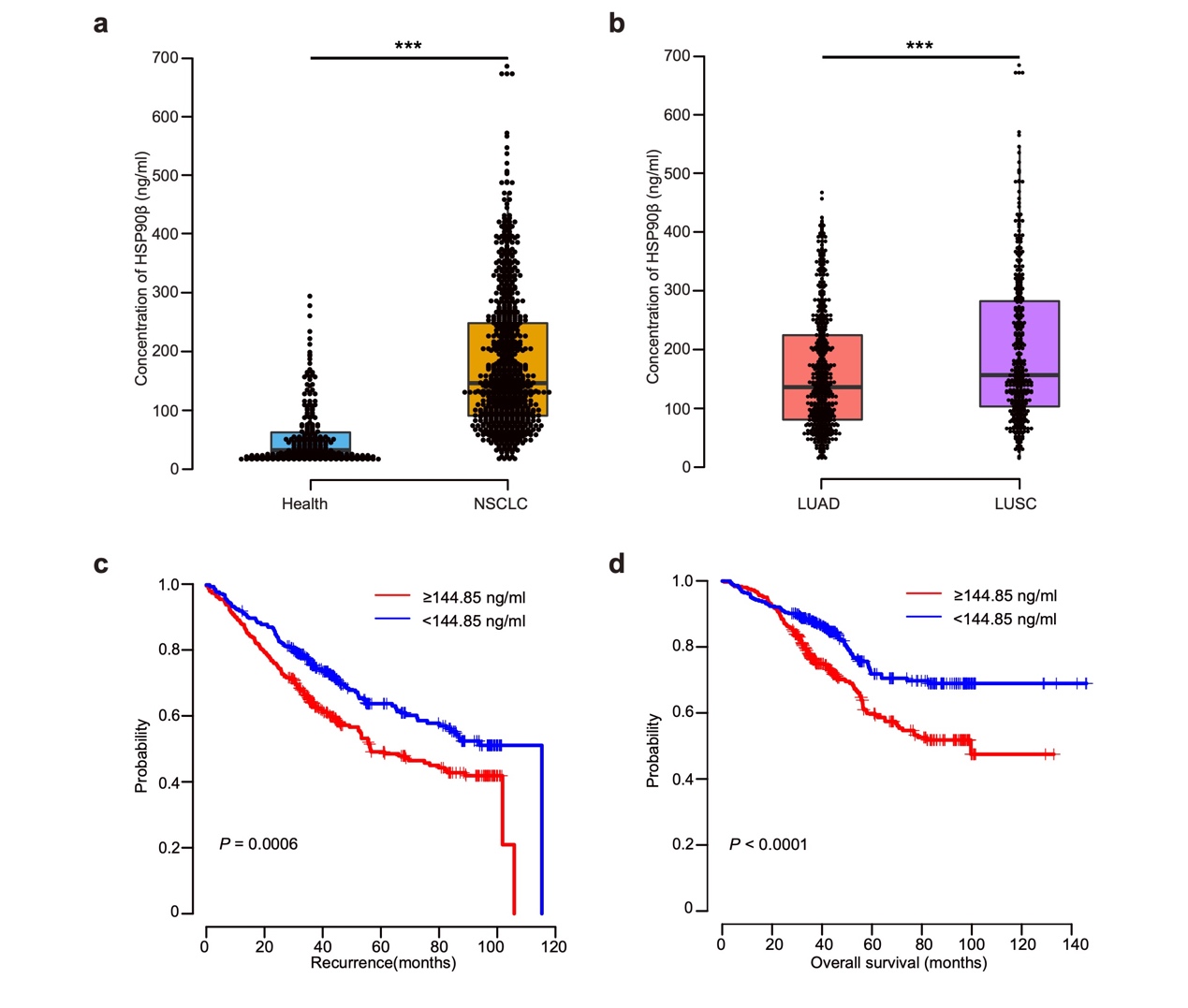


Figure. S2.

**Diagnostic impact and predictive value of the plasma levels of HSP90****β in NSCLC.**

**a** Expression of HSP90β in the plasma of NSCLC patients and healthy controls (Healthy: healthy control, n = 282; NSCLC: non-small-cell lung cancer, n = 870). **b** Expression of HSP90β in plasma of cancer patients between the LUAD and LUSC (LUAD: lung adenocarcinoma, n =499; LUSC: lung squamous cell carcinoma, n =371). **c** DFS curve of the 733 patients based on the median plasma level of HSP90β. **d** OS curve of the 733 patients based on the median plasma level of HSP90β. AUC: Area under the ROC curve. The 95% CI is indicated in parentheses. ***, *P* < 0.001.

**
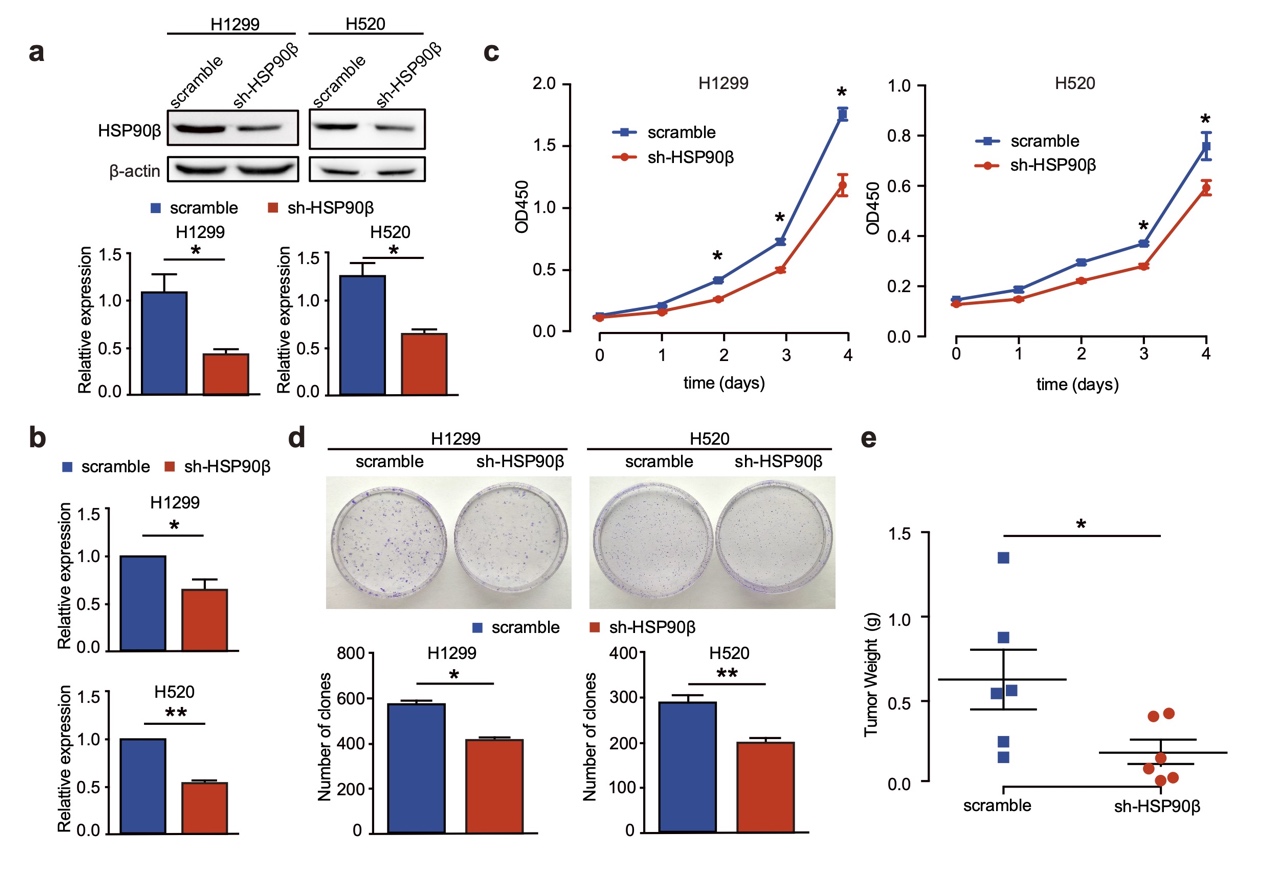
**

Figure. S3.

**HSP90β knockdown affected proliferation and the cell cycle as well as apoptosis in lung cancer cell lines.** **a** Western blot results following HSP90β knockdown in H1299 and H520 cells (upper panel shows the Western blot, lower panel shows the statistical column diagram of relative expression). **b** Expression of HSP90β for qPCR in cell lines H1299 and H520 after knockdown of HSP90β. **c** Proliferation curve of H1299 and H520 cells. **d** Clony formation of H1299 and H520 after knowndown of HSP90β (left panel shows the image of clones in cell lines, right panel shows the statistical number of clones in cell lines). **e** Tumor weight for the two groups of nude mice. *, *P* < 0.05. **, *P* < 0.01.

**
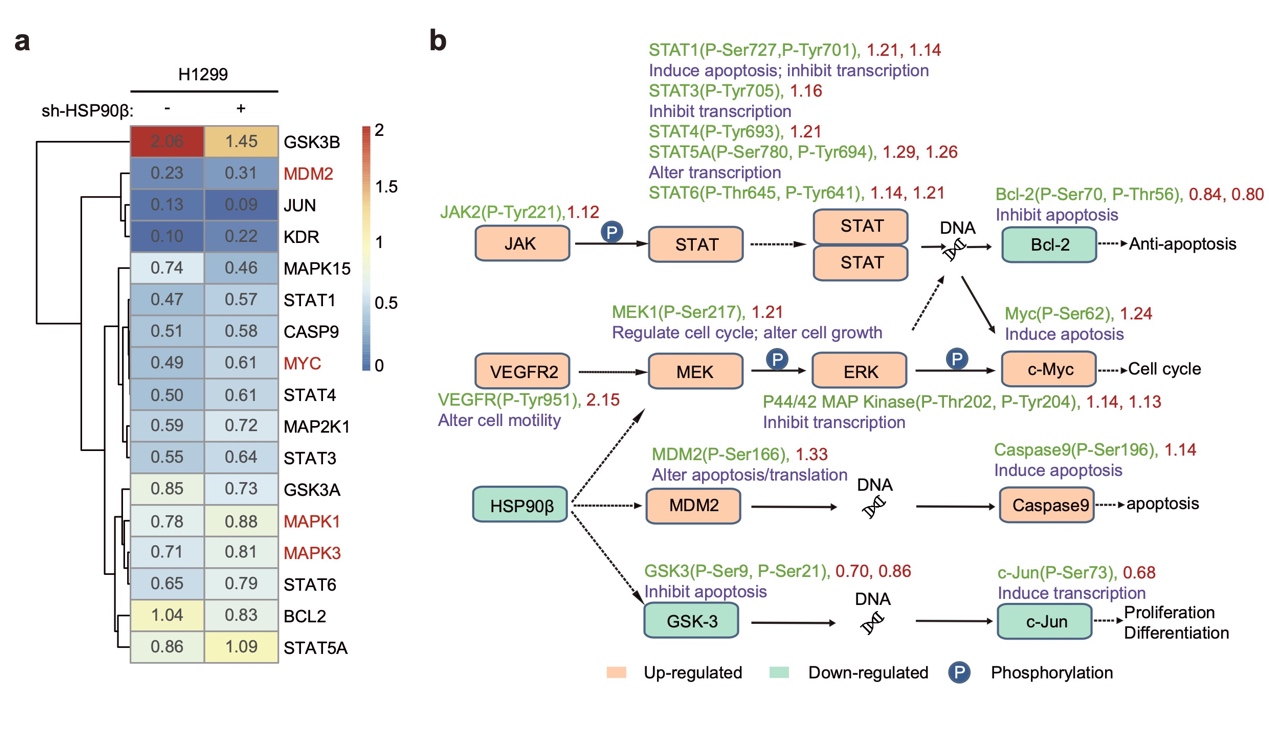
**

Figure. S4.

**HSP90β affected the phosphorylation pathways of several key proteins involved in apoptosis and the cell cycle in lung cancer cells.** **a** Heat map of 17 differentially phosphorylated proteins involved in the cell cycle and apoptosis. **b** Detailed differential signal pathways through the phospho-antibody array (The red numbers in the picture represents the fold change between the control and the HSP90β knockdown cell lines, functional annotations were listed in purple below the differential proteins).

**Table S6**: Correlation between HSP90β protein concentration and clinical information from the NSCLC

| **Variable** | **Number**  **n = 870** | **HSP90β levels (ng/ml)** | | | **P Value** |
| --- | --- | --- | --- | --- | --- |
|  |  | **Mean ± SD** | **Range** | **Median** |  |
| Age（y） |  |  |  |  | 0.5589 |
| ≤ 60 | 452 | 176.06 ± 114.79 | 14.93 - 673.49 | 143.34 |  |
| > 60 | 418 | 181.47 ± 117.09 | 15.54 - 686.28 | 146.90 |  |
| Gender |  |  |  |  | 0.0541 |
| Male | 564 | 184.41 ± 119.69 | 14.93 - 686.28 | 149.07 |  |
| Female | 306 | 168.06 ± 107.84 | 16.83 - 673.49 | 133.43 |  |
| Stage |  |  |  |  | 0.1332 |
| I | 404 | 174.06 ± 118.92 | 18.21 - 686.28 | 140.71 |  |
| II | 181 | 191.21 ± 115.60 | 16.66 - 520.62 | 161.57 |  |
| III-IV | 276 | 176.16 ± 110.64 | 14.93 - 673.27 | 144.07 |  |
| NA | 9 |  |  |  |  |
| Lymph node metastasis |  |  |  |  | 0.5255 |
| Yes | 384 | 178.46 ± 110.51 | 15.54 - 673.27 | 148.33 |  |
| No | 458 | 178.42 ± 120.33 | 14.93 - 686.28 | 142.56 |  |
| NA | 28 |  |  |  |  |
| Pathological types |  |  |  |  | <0.0001 |
| LUAD | 499 | 161.88 ± 100.65 | 14.93 - 468.91 | 136.60 |  |
| LUSC | 371 | 201.22 ± 130.39 | 15.54 - 686.28 | 157.12 |  |
| Differentiation |  |  |  |  | 0.2331 |
| Well | 94 | 158.71 ± 95.26 | 35.31 - 421.15 | 131.64 |  |
| Moderate | 462 | 184.89 ± 119.07 | 16.83 - 673.49 | 153.48 |  |
| Poor | 286 | 180.61 ± 117.95 | 14.93 - 686.28 | 144.31 |  |
| NA | 28 |  |  |  |  |
| Smoking status |  |  |  |  | 0.03 |
| Yes | 483 | 185.58 ± 117.83 | 16.66 - 686.28 | 148.27 |  |
| No | 381 | 169.47 ± 111.82 | 14.93 - 673.49 | 139.92 |  |
| NA | 6 |  |  |  |  |
| Family history |  |  |  |  | 0.0491 |
| Yes | 142 | 160.63 ± 106.25 | 16.66 - 673.49 | 128.7 |  |
| No | 718 | 181.69 ± 116.70 | 14.93 - 686.28 | 150.23 |  |
| NA | 10 |  |  |  |  |

**Table S7:** Differentially phosphorylated sites

| **Name** | **Gene Symbol** | **Swiss Prot** | **scramble_Phos/Unphos** | **sh HSP90β_Phos/Unphos** | **sh HSP90β_VS_scramble** |
| --- | --- | --- | --- | --- | --- |
| 4E-BP1 (Phospho-Thr36) | EIF4EBP1 | Q13541 | 0.62 | 0.91 | 1.48 |
| BAD (Phospho-Ser136) | BAD | Q92934 | 0.77 | 0.86 | 1.13 |
| BCL-2 (Phospho-Ser70) | BCL2 | P10415 | 0.99 | 0.83 | 0.84 |
| BCL-2 (Phospho-Thr56) | BCL2 | P10415 | 1.04 | 0.83 | 0.80 |
| Beta-Catenin (Phospho-Ser37) | CTNNB1 | P35222 | 0.56 | 0.77 | 1.39 |
| Beta-Catenin (Phospho-Thr41/Ser45) | CTNNB1 | P35222 | 0.51 | 0.64 | 1.25 |
| BRCA1 (Phospho-Ser1423) | BRCA1 | P38398 | 0.25 | 0.30 | 1.22 |
| BRCA1 (Phospho-Ser1524) | BRCA1 | P38398 | 0.54 | 0.73 | 1.34 |
| Caspase 9 (Phospho-Ser196) | CASP9 | P55211 | 0.51 | 0.58 | 1.14 |
| Caveolin-1 (Phospho-Tyr14) | CAV1 | Q03135 | 0.70 | 0.80 | 1.14 |
| cdc25C (Phospho-Ser216) | CDC25C | P30307 | 0.46 | 0.54 | 1.17 |
| Chk1 (Phospho-Ser345) | CHEK1 | O14757 | 0.45 | 0.53 | 1.18 |
| c-Jun (Phospho-Ser73) | JUN | P05412 | 0.13 | 0.09 | 0.68 |
| c-Kit (Phospho-Tyr721) | KIT | P10721 | 0.63 | 0.55 | 0.86 |
| elF4E (Phospho-Ser209) | EIF4E | P06730 | 0.69 | 0.79 | 1.14 |
| Elk-1 (Phospho-Ser383) | ELK1 | P19419 | 1.19 | 0.88 | 0.74 |
| ERK8 (Phospho-Thr175/Tyr177) | MAPK15 | Q8TD08 | 0.74 | 0.46 | 0.62 |
| FAK (Phospho-Tyr925) | PTK2 | Q05397 | 0.53 | 0.63 | 1.18 |
| GSK3 alpha (Phospho-Ser21) | GSK3A | P49840 | 0.85 | 0.73 | 0.86 |
| GSK3 beta (Phospho-Ser9) | GSK3B | P49841 | 2.06 | 1.45 | 0.70 |
| ICAM-1 (Phospho-Tyr512) | ICAM1 | P05362 | 0.65 | 0.83 | 1.28 |
| IkB-alpha (Phospho-Ser32/Ser36) | NFKBIA | P25963 | 5.20 | 3.74 | 0.72 |
| IkB-alpha (Phospho-Tyr42) | NFKBIA | P25963 | 0.52 | 0.61 | 1.17 |
| Integrin beta-3 (Phospho-Tyr773) | ITGB3 | P05106 | 0.79 | 0.66 | 0.84 |
| JAK2 (Phospho-Tyr221) | JAK2 | O60674 | 0.67 | 0.75 | 1.12 |
| Keratin 18 (Phospho-Ser33) | KRT18 | P05783 | 0.51 | 0.66 | 1.29 |
| MDM2 (Phospho-Ser166) | MDM2 | Q00987 | 0.23 | 0.31 | 1.33 |
| MEK1 (Phospho-Ser217) | MAP2K1 | Q02750 | 0.59 | 0.72 | 1.21 |
| mTOR (Phospho-Ser2448) | MTOR | P42345 | 0.66 | 0.88 | 1.33 |
| Myc (Phospho-Ser62) | MYC | P01106 | 0.49 | 0.61 | 1.24 |
| NFkB-p65 (Phospho-Ser529) | RELA | Q04206 | 0.66 | 0.75 | 1.15 |
| p44/42 MAP Kinase (Phospho-Thr202) | MAPK3 | P27361 | 0.71 | 0.81 | 1.14 |
| p44/42 MAP Kinase (Phospho-Thr202) | MAPK1 | P28482 | 0.71 | 0.81 | 1.14 |
| p44/42 MAP Kinase (Phospho-Tyr204) | MAPK3 | P27361 | 0.78 | 0.88 | 1.13 |
| p44/42 MAP Kinase (Phospho-Tyr204) | MAPK1 | P28482 | 0.78 | 0.88 | 1.13 |
| p53 (Phospho-Ser315) | TP53 | P04637 | 0.08 | 0.15 | 1.79 |
| PDK1 (Phospho-Ser241) | PDPK1 | O15530 | 1.28 | 0.81 | 0.63 |
| Pyk2 (Phospho-Tyr402) | PTK2B | Q14289 | 0.61 | 0.75 | 1.23 |
| Rac1/cdc42 (Phospho-Ser71) | RAC1 | P63000 | 0.59 | 0.68 | 1.16 |
| Rel (Phospho-Ser503) | REL | Q04864 | 1.02 | 0.87 | 0.85 |
| Shc (Phospho-Tyr349) | SHC1 | P29353 | 0.22 | 0.34 | 1.51 |
| SHP-2 (Phospho-Tyr580) | PTPN11 | Q06124 | 0.50 | 0.65 | 1.31 |
| Src (Phospho-Tyr418) | SRC | P12931 | 0.20 | 0.22 | 1.12 |
| STAT1 (Phospho-Ser727) | STAT1 | P42224 | 0.47 | 0.57 | 1.21 |
| STAT1 (Phospho-Tyr701) | STAT1 | P42224 | 0.61 | 0.69 | 1.14 |
| STAT3 (Phospho-Tyr705) | STAT3 | P40763 | 0.55 | 0.64 | 1.16 |
| STAT4 (Phospho-Tyr693) | STAT4 | Q14765 | 0.50 | 0.61 | 1.21 |
| STAT5A (Phospho-Ser780) | STAT5A | P42229 | 0.07 | 0.09 | 1.29 |
| STAT5A (Phospho-Tyr694) | STAT5A | P42229 | 0.86 | 1.09 | 1.26 |
| STAT6 (Phospho-Thr645) | STAT6 | P42226 | 0.32 | 0.36 | 1.14 |
| STAT6 (Phospho-Tyr641) | STAT6 | P42226 | 0.65 | 0.79 | 1.21 |
| Tau (Phospho-Ser404) | MAPT | P10636 | 0.36 | 0.45 | 1.25 |
| VEGFR2 (Phospho-Tyr951) | KDR | P35968 | 0.10 | 0.22 | 2.15 |
